# Supplementary material for: Physical Activity, Body Mass Index, and Bullying in Higher Education: A Comparative Analysis of Students with and Without Structured Sports Training
Source: Healthcare (Basel). 2025 Sep 15;13(18):2304. doi: 10.3390/healthcare13182304 (PMC12469962; doi:10.3390/healthcare13182304)
Supplement: Supplementary file 1 [file healthcare-13-02304-s001.zip › healthcare-3819951-supplementary.pdf]

Supplementary Material

„PHYSICAL ACTIVITY, BODY MASS INDEX AND BULLYING IN THE UNIVERSITY ENVIRONMENT:
A COMPARATIVE ANALYSIS BETWEEN STUDENTS WITH AND WITHOUT SPORTS TRAINING"

Raluca Mijaica[1]* and **Lorand Balint**[1]

[1] *Faculty of Physical education and mountain sports, Department of physical education and special motricity, Transylvania University of Brașov, Str. Universității No. 1, 500068 Brașov, Romania*
raluca_mijaica@unitbv.ro (R.M.); *lbalint@unitbv.ro* (L.B.)
*Corresponding author: **raluca_mijaica@unitbv.ro**

Contents

- Informed Consent and Study Introduction
- Physical Activity Questionnaire (Adapted PAQ-A)
- Bullying and Cyberbullying Questionnaire
- Body Mass Index (BMI) Measurement Form
- Methodology for Data Coding and Processing
- Summary Data Collection Sheet (Blank and Example)
- Completion Instructions for Faculty Coordinators

TRANSYLVANIA UNIVERSITY OF BRAȘOV

FACULTY OF PHYSICAL EDUCATION AND MOUNTAIN SPORTS

STUDY ON PHYSICAL ACTIVITY, BODY MASS INDEX AND BULLYING BEHAVIORS AMONG FIRST-YEAR STUDENTS (2023–2024)

Participant code: ________ (to be completed by the investigator/academic staff)

INFORMATION AND INFORMED CONSENT

Dear student,

You are invited to participate in a scientific study conducted by Transylvania University of Brașov. The aim of this research is to investigate the level of physical activity, body weight status (BMI), and relational behaviors (bullying and cyberbullying) among first-year university students.

The data collection process will take place between January 08–19, 2024, within your faculty, under the supervision of the research team.

What does your participation involve?

- Completion of two questionnaires (physical activity and bullying);
- Measurement of height and weight, used to calculate the Body Mass Index (BMI).

Your data will be anonymous and confidential

- You will not be asked to provide your name or any personal identifiers;
- All information will be coded and processed solely for scientific purposes, in accordance with the European data protection regulations (GDPR 2016/679);
- Participation is voluntary – you may withdraw at any time without consequences.

By completing this instrument set, you are providing your informed consent to participate in the study.

Thank you for your valuable contribution!

PARTICIPANT IDENTIFICATION DATA

Student code: __________ (to be completed by the researcher at the time of distribution)
• **Sex:** ☐ M ☐ F **Age (in full years):** _______

Select your faculty (circle the corresponding code):

Code	Faculty	Code	Faculty
01	Faculty of Product Design and Environment	10	Faculty of Mathematics and Computer Science
02	Faculty of Mechanical Engineering	11	Faculty of Medicine
03	Faculty of Materials Science and Engineering	12	Faculty of Psychology and Education Sciences
04	Faculty of Letters	13	Faculty of Sociology and Communication
05	Faculty of Music	14	Faculty of Food and Tourism
06	Faculty of Economic Sciences and Business Administration	15	Faculty of Civil Engineering
07	Faculty of Electrical Engineering and Computer Science	16	Faculty of Furniture Design and Wood Engineering
08	Faculty of Technological Engineering and Industrial Management	17	Faculty of Forestry and Forest Exploitation
09	Faculty of Law	18*	Faculty of Physical Education and Mountain Sports

*If you selected **code 18** (Faculty of Physical Education and Mountain Sports), please tick the study program you are enrolled in:

- ☐ **EFS** – Physical Education and Sports
- ☐ **SPM** – Sport and Motor Performance
- ☐ **KMS** – Kinesiotherapy and Special Motricity

• **Year of study:** I **Semester:** I **Date of completion:** ____ / ____ / 2024

Physical Activity Questionnaire (Adapted PAQ-A)

Reference period: October 02, 2023 – January 19, 2023 (including holidays)
All questions refer to your behavior during this period.

Student Code: ________

Please select only **one option** for each question, choosing the one that best describes your behavior during the indicated period.

Evaluation scale:

1 = Very low | 2 = Low | 3 = Moderate | 4 = High | 5 = Very high

**No.**	**Question**	**1** ☐	**2** ☐	**3** ☐	**4** ☐	**5** ☐
1	How intensely did you physically engage in motor activities organized by your faculty (e.g., courses, labs, applied sports)?	☐	☐	☐	☐	☐
2	On average, how frequently did you engage in physical activities outside your faculty (e.g., running, gym, sports, hiking, dancing, etc.)? (Think about a typical week over the past 4 months)	☐	☐	☐	☐	☐
3	How would you evaluate your physical engagement during breaks between classes and your free time on campus (walking, using sports facilities, recreation)?	☐	☐	☐	☐	☐
4	During weekends, how often did you engage in physical activities (e.g., long walks, sports games, hiking, gym, etc.)? (Consider the average over both weekend days)	☐	☐	☐	☐	☐
5	How often did you participate in informal physical activities with friends or colleagues (games, workouts, sports outings, etc.)?	☐	☐	☐	☐	☐
6	How would you describe a typical week during this period in terms of your overall level of physical activity? (Exclude atypical weeks: illness, exams, trips, etc.)	☐	☐	☐	☐	☐
7	In a typical week during this period, on how many days were you physically active for at least 60 minutes per day? (Include any type of moderate or vigorous activity)	☐	☐	☐	☐	☐
8	Compared to your peers, how would you rate your general level of physical activity over the past 4 months? (This is a subjective evaluation)	☐	☐	☐	☐	☐

Additional question ***(methodological purpose – not included in the final score):***
In the past 3–4 years, have you consistently practiced organized sports (at least 3 times/week), being registered with or affiliated to a sports club?

○ Yes ○ No

Relational Behavior Questionnaire

(Bullying and Cyberbullying)

Reference period: October 02, 2023 – January 19, 2024 (including holidays)
All questions refer to situations within the university environment, in relation to other students.

Student Code: ________

Instructions:

For each of the following statements, check the frequency with which you **experienced** the situation (as a *victim*) or **engaged** in the behavior (as a *perpetrator*) during the indicated period.

Response scale:

1 = Never | 2 = Rarely | 3 = Sometimes | 4 = Often | 5 = Very often

I. Traditional Victimization (face-to-face)

No.	Statement	1 ☐	2 ☐	3 ☐	4 ☐	5 ☐
1	I felt repeatedly excluded from peer groups.	☐	☐	☐	☐	☐
2	I was teased, humiliated, or verbally insulted by other students.	☐	☐	☐	☐	☐
3	I was mocked or subjected to sarcastic remarks in front of others.	☐	☐	☐	☐	☐
4	I was the target of rumors spread about me.	☐	☐	☐	☐	☐
5	I was deliberately ignored or treated with hostility by peers.	☐	☐	☐	☐	☐

II. Traditional Aggression (face-to-face)

No.	Statement	1 ☐	2 ☐	3 ☐	4 ☐	5 ☐
6	I spread rumors about other students.	☐	☐	☐	☐	☐
7	I mocked or verbally insulted other students.	☐	☐	☐	☐	☐
8	I physically or verbally intimidated a peer to control them.	☐	☐	☐	☐	☐
9	I deliberately excluded a peer from a group or activity.	☐	☐	☐	☐	☐
10	I made repeated mean jokes or comments about a peer.	☐	☐	☐	☐	☐

III. Cyber Victimization (online)

No.	Statement	1 ☐	2 ☐	3 ☐	4 ☐	5 ☐
11	I received offensive or hostile messages online (chat, groups, etc.).	☐	☐	☐	☐	☐

12	I was excluded from online peer groups.	☐	☐	☐	☐	☐
13	I was criticized or ridiculed in comments on social media or student platforms.	☐	☐	☐	☐	☐
14	Personal information or images of me were posted online without my consent.	☐	☐	☐	☐	☐
15	I was the target of hostile or mocking reactions in digital university spaces.	☐	☐	☐	☐	☐

IV. Cyber Aggression (online)

**No.**	**Statement**	**1** ☐	**2** ☐	**3** ☐	**4** ☐	**5** ☐
16	I sent offensive messages to a peer via the Internet.	☐	☐	☐	☐	☐
17	I publicly criticized or mocked a peer in an online group.	☐	☐	☐	☐	☐
18	I deliberately blocked or excluded a peer from a digital group.	☐	☐	☐	☐	☐
19	I shared images or information about a peer without their consent.	☐	☐	☐	☐	☐
20	I participated in online discussions with the intent to isolate or ridicule a peer.	☐	☐	☐	☐	☐

Body Mass Index (BMI) Assessment Sheet

Transilvania University of Brașov – Faculty of Physical Education and Mountain Sports
Assessment period: January 08–19, 2024

This form is part of the investigation package regarding physical activity, body weight status, and relational behaviors among first-year students.
Please fill in the following data based on the measurements conducted with the support of the research team.

Student Code: ________________________

Height (measured using stadiometer): __________ cm

Weight (measured using Tanita MC-780 MA analyzer): __________ kg

Body Mass Index (BMI) – value displayed on the device screen: ____________

You do **not** need to interpret your BMI result – it will be confidentially analyzed by the research team.

Next step:
RETURN THIS FORM TOGETHER WITH THE COMPLETED QUESTIONNAIRES.

Thank you for your participation!

Methodology for Data Coding and Processing

This methodological guide is intended to ensure consistent processing of data collected through the assessment instruments used in the study on physical activity, body mass index (BMI), and relational behaviors (bullying and cyberbullying) among first-year students.

1. Participant Coding

Each participant was assigned a **unique alphanumeric code** according to the following format:

For most faculties (codes 01–17):

XXYZZZ

- **XX** = faculty code (e.g., 05 = Music, 17 = Forestry)
- **Y** = participant's sex (F = female, M = male)
- **ZZZ** = sequential number within that faculty

Examples:

- 05F014 = Female student no. 14 from the Faculty of Music
- 17M007 = Male student no. 7 from the Faculty of Forestry

For the Faculty of Physical Education and Mountain Sports (code 18):

Due to the three distinct study programs, an extended coding structure was used:

18P-Y-ZZZ

- **18** = faculty code
- **P** = program of study:
  - **E** = Physical Education and Sports (EFS)
  - **S** = Sport and Motor Performance (SPM)
  - **K** = Kinesiotherapy and Special Motricity (KMS)
- **Y** = sex (F = female, M = male)
- **ZZZ** = sequential number within that program

Examples:

- 18E-F011 = Female student no. 11, enrolled in EFS
- 18S-M003 = Male student no. 3, enrolled in SPM
- 18K-F020 = Female student no. 20, enrolled in KMS

This differentiated structure allows for comparative analysis across programs within the Faculty of Physical Education and Mountain Sports, in line with the study design.

2. Physical Activity Data Processing (Adapted PAQ-A)

The questionnaire includes:

- **8 scored items** used to compute the composite score

- **1 additional filtering question** on prior organized sport participation (not scored)

Coding:

- Each response is scored from **1 to 5** using a Likert scale
- The overall PAQ-A score is the **arithmetic mean** of the 8 scored items

Exclusion criteria for analysis:

- Students who answered **“Yes”** to the additional question (i.e., practiced organized sport regularly—at least 3 times/week—for the past 3–4 years) are excluded from comparative analyses between **non-sports faculties**.

3. BMI Calculation and Classification

For each participant:

- **Body weight** was measured using the Tanita MC-780 MA analyzer
- **Height** was measured using a professional stadiometer and manually entered into the Tanita device
- The device automatically computed the BMI value

BMI Classification (WHO criteria):

- < 18.5 = Underweight
- 18.5–24.9 = Normal weight
- 25.0–29.9 = Overweight
- ≥ 30.0 = Obese

BMI values were retrieved from the printed Tanita report and recorded in the individual form. **No interpretation** was requested from participants.

4. Relational Behavior Data Processing (Bullying and Cyberbullying)

The questionnaire includes **20 items**, grouped into **4 subscales**:

Subscale	No. of Items	Type of Behavior
I. Traditional Victimization	5	Student as victim, face-to-face context
II. Traditional Aggression	5	Student as aggressor, face-to-face context
III. Cyber Victimization	5	Student as victim, online context
IV. Cyber Aggression	5	Student as aggressor, online context

Coding:

- Items are scored from **1 (“Never”) to 5 (“Very often”)**
- Each subscale totals between **5 and 25 points**, by summing the relevant 5 items

Typological Classification:
Based on subscale scores, students can be categorized as:

- **Uninvolved** – very low or zero scores across all four subscales
- **Victims only** – high scores in I or III, but not II or IV
- **Aggressors only** – high scores in II or IV, but not I or III
- **Victim + Aggressor** – high scores in both victim and aggressor subscales
- **Involved** – sum of all those engaged in any role

5. Summary Data Collection Sheet (SPSS or Excel)

To facilitate accurate data entry into SPSS or Excel, each faculty coordinator must complete one standardized sheet per student (see model below).

The sheet must include:

- Demographic and administrative data
- Raw item scores from the PAQ-A questionnaire (items 1–8)
- Calculated average PAQ-A score
- BMI data
- Total scores for each relational behavior subscale (bullying and cyberbullying)

Completion format:

- Filled manually (printed version)
- Digital entry will be done by the designated data operator

The following are included below:

- A **blank data sheet** (to be completed for each student)
- A **completed example sheet** (fictional data, for reference)

Faculty coordinators must:

- Fill in each field clearly and legibly, following form instructions
- Verify data accuracy based on the three sources: PAQ-A questionnaire, BMI sheet, bullying questionnaire
- Submit completed forms physically or scanned (as per protocol)

It is essential that:

- PAQ-A item scores (1–8) and bullying subscale totals are entered **exactly as recorded**
- BMI value is copied directly from the Tanita device
- BMI category is **ticked accordingly**

DATA COLLECTION SHEET – BLANK TEMPLATE

- **Student Code: ___________________________**
- **Sex:** ☐ F ☐ M
- **Age (years): _______**
- **Faculty (code): _______**
- **Program (if code 18):** ☐ EFS ☐ SPM ☐ KMS
- **Prior organized sport:** ☐ Yes ☐ No
- **Date of completion: ____** / **____** / 2024

PAQ-A Questionnaire – Item Scores (1–8):

PAQ_1 PAQ_2 PAQ_3 PAQ_4 PAQ_5 PAQ_6 PAQ_7 PAQ_8
___ ___ ___ ___ ___ ___ ___ ___ ___

- **Total PAQ-A Score (mean): __________**

BMI Evaluation:

- **Height:** ______ cm **Weight:** ______ kg
- **Displayed BMI: ________**
- **BMI Category:** ☐ Underweight ☐ Normal ☐ Overweight ☐ Obese

Relational Behavior – Subscale Scores (sum):

BT_victim BT_aggressor CB_victim CB_aggressor
____ ____ ____ ____

DATA COLLECTION SHEET – COMPLETED EXAMPLE (FICTITIOUS)

- **Student Code:** 18K-F023
- **Sex:** ☒ F ☐ M
- **Age (years):** 20
- **Faculty (code):** 18
- **Program:** ☐ EFS ☐ SPM ☒ KMS
- **Prior organized sport:** ☒ Yes ☐ No
- **Date of completion:** 17 / 01 / 2024

PAQ-A Questionnaire – Item Scores (1–8):

PAQ_1	PAQ_2	PAQ_3	PAQ_4	PAQ_5	PAQ_6	PAQ_7	PAQ_8
4	5	4	5	3	4	5	4

Total PAQ-A Score (mean): 4.25

BMI Evaluation:

- **Height:** 166 cm **Weight:** 63.0 kg
- **Displayed BMI:** 22.8 **BMI Category:** ☒ Normal

Relational Behavior – Subscale Scores (sum):

BT_victim	BT_aggressor	CB_victim	CB_aggressor
7	6	8	5

This methodological section is intended **for the research team only** and should **not** be distributed to participants!

Instructions for Faculty Coordinators

To ensure accurate and consistent data collection, please follow the steps below when completing the summary sheet for each student:

What you need to do:

1. **Fill in all fields on the sheet**, using the information provided in the three completed instruments:
   - Physical Activity Questionnaire (PAQ-A);
   - Body Mass Index (BMI) Measurement Sheet;
   - Bullying and Cyberbullying Questionnaire.
2. **Write clearly and legibly** (preferably in pen, not pencil).
3. **Enter the exact values as recorded**, without adjustments or rounding:
   - For PAQ-A: transfer the raw scores for items 1–8 as given;
   - Calculate and record the average total PAQ-A score (e.g., 3.75);
   - Copy the BMI value directly from the Tanita device (e.g., 22.8);
   - Tick the appropriate BMI category according to WHO standards;
   - For bullying/cyberbullying: sum the scores for each subscale (5 items × 4 dimensions) and enter the totals.
4. **For the Faculty of Physical Education and Mountain Sports (code 18):**
   - Make sure to check the appropriate study program (EFS, SPM, or KMS);
   - Participant codes must include the program letter (e.g., 18K-F012).
5. **Verify completeness before submission:**
   - No fields should be left blank;
   - If a student is absent or refuses to participate, record this in a separate table—not on the form.
6. **Submitting the forms:**
   - Return all completed forms in physical or scanned format, according to the instructions provided by the data coordinator;
   - Do not keep local copies of the forms to ensure data confidentiality.

This supplementary material provides detailed information on the instruments, procedures, and coding methodology used in the present study. It is made available to ensure transparency and reproducibility of the research, in line with MDPI Research Data Policies and the FAIR data principles.
